# Evaluation of Community Involvement and Development in an Orthopedic Hospital

**DOI:** 10.3390/healthcare12131286

**Published:** 2024-06-27

**Authors:** Flaviu Moldovan, Liviu Moldovan

**Affiliations:** 1 Orthopedics—Traumatology Department, Faculty of Medicine, “George Emil Palade” University of Medicine, Pharmacy, Science, and Technology of Targu Mures, 540142 Targu Mures, Romania; 2 Faculty of Engineering and Information Technology, “George Emil Palade” University of Medicine, Pharmacy, Science, and Technology of Targu Mures, 540142 Targu Mures, Romania; liviu.moldovan@umfst.ro

**Keywords:** community involvement and development, sustainability, orthopedics, reference framework, healthcare facility, assessment

## Abstract

Improving healthcare requires appropriate community involvement supported by appropriate partner engagement methods. This research aims to develop a complex tool for evaluating the social responsibility of health facilities regarding community involvement and development. We developed areas of a new reference framework for the sustainability of healthcare organizations, which includes the area of community involvement and expansion. It is made up of nine indicators. These were designed using the most representative activities reported by hospitals around the world. Their testing was conducted in an orthopedic emergency hospital. The designed indicators are community engagement actions; the interventions’ content adapted to the community; partnership and networking; the involvement of volunteers and training networks; the involvement and participation of professional associations; community-involved local opinion leaders; satisfaction with partnerships; initiatives together with the community; and educational visits. The testing and validation of health practices of the indicators highlighted their adequacy with the proposed purpose of the research and the promotion of sustainable development. We have also verified their compatibility with the requirements of national hospital accreditation legislation and the European framework for quality assurance in hospitals.

## 1. Introduction

With the increase in the technological complexity, sophistication, and centralization of national health services, most of these services have come under the exclusive responsibility of professional healthcare staff. However, for effective healthcare services to be extended to most of the population, community involvement is needed. From this perspective, there is a need for leadership and collaboration with decision makers, but also the creation of appropriate tools. They enable the optimization of benefits for medical staff, patients, and the population that would benefit from a wider medical practice [1].

The World Health Organization, through the Alma-Ata declaration [2] and more recently through the Astana declaration [3], developed a strategy with which to revolutionize the practice of healthcare and health progress. This has as an essential element the promotion of better and effective community participation in the services and structures designed to ensure the health of the population. Public organizations support patient-centered care and shared decision making. Several health and research institutions have established patient advisory boards. In Germany, although efforts have been made to introduce shared decision making into the practice of healthcare or physical therapy providers, many steps are needed for its reliable implementation in routine care [4]. Moumjid et al. [5] shows that in France the implementation of shared decision making with community support in clinical practice and real-life health environments requires a series of tools and dedicated human resources, clinical decision aids, clinical guidelines, etc. The need for community partnership to support patient-centered care processes and shared decision making is also emphasized by other researchers from Israel [6], Iran [7], Denmark [8], Malaysia [9], Canada [10], etc. in recently published papers.

Through community involvement, the planning, development, and implementation of healthcare services has improved [11]. With the support of remarkably diverse conceptualizations and methodological approaches, a wide range of public involvement methods have been developed [12]. However, there is a lack of coherence in community involvement methods, which promotes uncertainty regarding the motivation, place, and time of applying these methods [13]. Community participation is a principle of primary healthcare. But due to numerous barriers, it has not been adequately institutionalized. Gholipour et al. [14] indicates some barriers: managerial approaches in the healthcare system, institutional obstacles, cultural barriers, community trust in the healthcare system, community perception, and the status of community participation programs.

Partnerships with patients, the community, and the public are imperative in improving healthcare [15]. But this requires changing individual (healthcare staff and managers), organizational (healthcare facilities), and system-level (collaboration between organizations, funding policies) behavior. A supporting factor is the training of health professionals. The barriers are the lack of time and resources for community involvement, finding partner institutions in projects, and a perceived lack of evidence on the impact and effectiveness of community involvement [16]. Heumann et al. [17] show that facilitating community and patient participation requires motivating patients for self-management, along with allocating appropriate resources; collecting patient requirements regarding the development of medical services; sharing and understanding patients’ health needs; and supporting individual and community networks of patients.

Sacks et al. [18] identified several frames of reference through which they highlighted the roles determined for organizations and community members, which remain relevant today as stated by Laurisz et al. [19]. Despite evidence that community involvement improves the effectiveness of many healthcare programs, communities can be better involved in the following areas: planning and goal setting; the implementation of healthcare programs; evaluation and continuous improvement; and incorporating activities into healthcare-system-strengthening frameworks.

Having as a starting point these controversies revealed by the scientific literature regarding community involvement and development, in the present study we formulated the following research questions:

RQ 1: What are the defining aspects of community involvement and development?

RQ 2: What are the good practices validated by representative international healthcare facilities and reported in the scientific literature that support the implementation of community involvement and development?

RQ 3: With the support of good practices collected from the scientific literature, what are the indicators that can be used to evaluate community involvement and development?

RQ 4: How can the indicators designed for the evaluation of community involvement and development be qualitatively and quantitatively evaluated so that their implementation is monitored?

In the next step, with the support of these research questions, we stated the objective of our study: the development of a complex tool for evaluating the social responsibility of healthcare facilities regarding community involvement and development. A secondary aim of the research is to ensure the compatibility of the new tool with other reference frameworks implemented in healthcare facilities. The novelty of the research results from the content and evaluation method of the indicators that make up the new evaluation tool.

## 2. Materials and Methods

In the research methodology we went through the following stages:

1. We developed the fields of the reference framework of the sustainability of healthcare facilities, in which we included the field of community involvement and development, according to the requirements of the social responsibility standard ISO 26000 [20];
2. We explored the specialized scientific literature and extracted the most representative activities reported by hospitals around the world that characterize community involvement and development. This is a source of the most up-to-date global information that is confirmed in practice [21];
3. With the support of the activities from the previous point, we designed the indicators for the evaluation of community involvement and development, a method that is supported by confirmed healthcare practices [22];
4. We established the methodology for evaluating the indicators and developed qualitative and quantitative grids for the evaluation of each indicator related to community involvement and development, with the support of which we can prioritize improvement measures [23];
5. We tested the feasibility of the indicators in practice at an emergency hospital in the orthopedic specialty and validated their contents, a method that confirms their reliability [24].

### 2.1. The Reference Framework Areas

**Figure 1:**
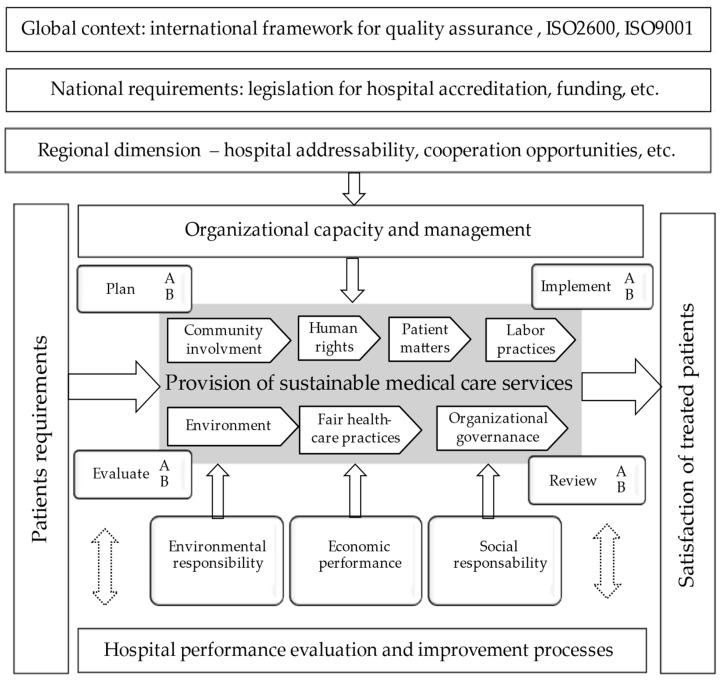
Succession and interaction of the areas of the Health-Sustainability (H-S) reference framework and the basic medical activities organized in the stages of the quality cycle: Plan—Design of medical assistance services, Plan-A—Hospital institutional accreditation, and Plan-B—Designing patient-oriented medical services; Implement—Provision of medical assistance services, Implement-A—Provision of medical services, and Implement-B—Patient transfer provision; Evaluation—Evaluation of medical assistance services, Evaluation-A—Local opinion leaders’ evaluation and involvement, and Evaluation-B—Satisfaction assessment for patients and staff; and Review—Medical assistance continuous improvement, Review-A—Staff self-assessment, and Review-B—Medical services innovation.

We established the areas of the reference framework for ensuring the quality and sustainability of healthcare facilities, Health-Sustainability (H-S), by including the three classic areas of sustainability: environmental, economic, and social. The last of these we developed in accordance with the requirements of the social responsibility standard ISO 26000 [20], which includes seven requirements. They have been adapted to the particularities of the medical field, as follows: organizational governance [25], human rights [26], labor practices [27], environment [28], fair healthcare services [29], patient matters [30], and community involvement and development. The entire system has the provision of sustainable medical assistance services as its basic processes, as can be seen in Figure 1.

We ordered the medical activities according to their content, in the succession of the quality cycle stages, by complying with the requisites of the ISO9001 quality assurance standard in the latest edition from 2015 [31]. Also, we customized the stages of the quality cycle to the specifics of the medical field, and corresponding to each stage of the quality cycle we foresaw two basic medical activities. In the first sequence, Plan—Design of medical assistance services, the basic medical activities are Plan-A—Hospital institutional accreditation, and Plan-B—Designing patient-oriented medical services. It is followed by Implement—Provision of medical assistance services, with the two activities Implement-A—Provision of medical services, and Implement-B—Patient transfer provision. The third sequence, Evaluation—Evaluation of medical assistance services, is composed of Evaluation-A—Local opinion leaders’ evaluation and involvement, and Evaluation-B—Satisfaction assessment for patients and staff. Finally, in the Review—Medical assistance continuous improvement stage, there are the activities Review-A—Staff self-assessment, and Review-B—Medical services innovation.

### 2.2. Proven Evidence of Community Involvement and Development Activities

Our research continued with the elaboration of the content of indicators related to the field of community involvement and development, as well as their evaluation grids.

**Figure 2:**
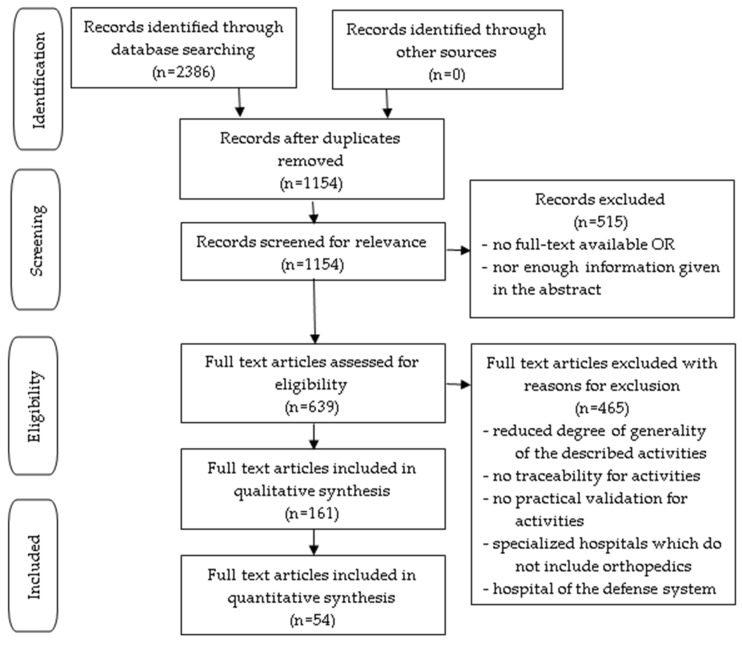
The systematic review PRISMA flow diagram, which details the number of records screened, and the full texts retrieved.

For this purpose, we acted in accordance with the Preferred Reporting Items for Systematic Reviews and Meta-Analyses (PRISMA) guidelines. We explored the medical literature from the most representative databases, such as PubMed, Web of Science, and EMBASE (OVID). The following keywords were applied to the search: (community involvement or community engagement actions or community adapted interventions) AND (partnership or networking or training networks) AND (volunteers or professional associations or community initiatives). Recent articles, mostly from the last 5–10 years, that presented new knowledge supported by evidence were preferred. In the case of articles that deal with the same aspect, we selected those that present a greater degree of applicability of the studied issue, but also better traceability. From here, we extracted the activities that support community involvement in the healthcare system, but also the barriers that do not support the adequate institutionalization of community participation, as can be seen in the PRISMA flow diagram (Figure 2).

We organized the obtained database according to the content’s classification in the stages of the quality cycle, which we present in the following subsections.

#### 2.2.1. Practices for the Design of Community Involvement and Development Activities

Community involvement in healthcare can be defined in many ways. Brunton et al. [32] identified three models that can ensure effective engagement. These are based on empowerment theory, collaboration between the community and health services based on different degrees of involvement, and respectively peer-led delivery. Following a meta-analysis, O’Mara-Eves et al. [33] show that community involvement creates a positive impact on health behaviors, social support outcomes, self-efficacy, and health outcomes. But the evidence on the economic effectiveness of community engagement interventions is weak. With these supports, different models of community involvement can be built that promote appropriate, effective, and sustainable initiatives.

Community involvement requires “meaningful citizen participation” that can only be achieved if a hospital has accessible, inclusive processes that support citizens [34]. For this, the training of medical staff with regard to the successful approach of community engagement actions is necessary [35]. However, some methodological constraints can limit the content of the information needed for the interventions and, with it, the key contents of the interventions [36]. Contextual differences between communities, in terms of demographics, social capital, and the availability of resources, must also be analyzed [37].

We used these medical practices to design the indicator PA7—Community engagement actions (having the content presented in Table A1). With their support, the basic medical activity of hospital institutional accreditation (Plan-A) is evaluated.

In the case of workers with musculoskeletal disorders, the effectiveness of community and workplace interventions aims to reduce absenteeism and avoid job loss. Palmer et al. [38] show that the benefits of these interventions are small, and employees must analyze the uncertainties through value judgments.

People with chronic pain can be helped by interdisciplinary pain rehabilitation programs to improve their health, manage their work, and reduce the length of sick leave. Interventions of this kind must be adapted to individual needs considering an employee’s situation [39]. Workers with musculoskeletal disorders who benefit from interventions at the workplace reduce their pain and improve their functional status, and the resumption of activity is facilitated [40]. However, the use of warm-up interventions in the workplace is not supported by conclusive evidence that they would alleviate work-related musculoskeletal disorders [41].

In sedentary workers, interventions to increase standing and walking durations reduce musculoskeletal symptoms in the short term [42]. Among the risk factors that cause back pain are waist circumference, watching television, and starting to smoke, but also intense physical activity [43].

We used these medical practices to design the indicator PB7—The interventions content adapted to the community (having the content presented in Table A3). With their support, the basic medical activity of designing patient-oriented medical services (Plan-B) is evaluated.

#### 2.2.2. Practices for the Provision of Medical Assistance Services

In forming a partnership, Raftery et al. [44] highlighted some essential requirements regarding the definition of a partnership’s mission, the analysis of the external environment, and the definition of management and communication systems, but also partners’ resources, in terms of skills and expertise. To these are added other networks in which the partners are included. Training effective leaders in managing partnerships requires training them in leadership through matchmaking mentoring schemes, the exchanging of best practices with opinion leaders, and an interactive website that enables national and international visibility [45].

Caring for aging patient populations with many chronic diseases requires appropriate patient health management by building patient-centered social networks. These are consistent with patient requirements and government policies for clinical effectiveness and economic efficiency [46]. Promoting the health of medically underserved communities can also be accomplished through faith-based organizations [47]. Partnerships created between the community and the academy generate benefits for patients because of the improvement of the research process [48].

The COVID-19 pandemic has disrupted the essential elements of traditional partnerships. To ensure their continuity, virtual partnership initiatives have rapidly been developed with the potential to reduce the gaps formed, to ensure bidirectionality and equity in partnerships [49].

We used these medical practices to design the indicator IA71—Partnership and networking (having the content presented in Table A5). With their support, the basic medical activity of the provision of medical services (Implement-A) is evaluated.

Volunteers support healthcare around the world. The efficiency of their integration into health systems requires motivation, education, goal setting, and a good connection with primary care as well as community resources [50]. Strategies for integrating community volunteers into the primary care environment require clarifying the roles and boundaries of volunteers, supporting efforts to connect volunteers with the primary care team they support, and training volunteers through role-playing [51].

But the integration of volunteers into the daily care of patients must be supported by hospital policies. For this, the following are necessary: a shared vision between the hospital and volunteer associations; developing integrated models of care that combine medical staff with volunteers; personalized continuing education of the volunteer network members; personalized education of the medical staff to value the services offered by volunteers; and adequate initial training of the volunteer network [52].

Kowalski et al. [53] show that working with networks of patients and their families has several benefits that are supported by interprofessional simulation courses delivered through training networks. Using available individual patient information, machine learning-based approaches can remotely predict metastases and survival chances of patients diagnosed with osteosarcoma [54]. Rangan et al. [55] highlighted the benefits of clinical trial networks in orthopedic surgery. These are conditional on adequate investment in testing infrastructure and multidisciplinary collaboration.

We used these medical practices to design the indicator IA72—Involvement of volunteers and training networks (with the content presented in Table A7). With their support, the basic medical activity of the provision of medical services (Implement-A) is evaluated.

Health professions are guided by codes of conduct based on the principles of responsibility, altruism, respect, excellence, ethics, communication, integrity, and justice. With this support, a common interprofessional framework can be established [56] and contribute to continuing medical education [57]. Likewise, professional healthcare associations must avoid funding auxiliary materials and food substitutes [58].

The primary issues of the professional association of emergency nurses are the prevention of trauma and injury, the provision of care to vulnerable populations, and the safety of patients [59]. The professional associations of orthopedic nurses evaluated their members by high scores on the perception of professional values, level of education and professional experience. They reflect a good understanding of how healthcare is delivered [60]. A potential way to improve the morale of orthopedic professionals is to strengthen the key elements that shape easy-going personality styles [61].

We used these medical practices to design the indicator IB7—Involvement and participation of professional associations (with the content presented in Table A9). With their support, the basic medical activity of patient transfer provision (Implement-B) is evaluated.

#### 2.2.3. Practices for the Evaluation of Medical Assistance Services

Using local opinion leaders to convey advanced medical norms and shape behavior is suggested by the social influence pattern of behavior change and the innovation diffusion theory [62]. Crane et al. [63] show that, by involving local opinion leaders in the community, health promotion campaigns are improved, without determining their effects on increasing the demand for evidence-based care. For critical access hospitals, opinion leaders working with large healthcare centers and community service organizations is an essential strategy as care delivery issues are addressed [64].

Opinion leaders are professionals perceived as credible in professional practice, who convey the best evidence regarding advanced medical practices to the community. They use informal teaching and educational community visits as methods [65]. Educational meetings promote the implementation of innovations and new knowledge. These have the effect of changing behavior and current practices within health systems, as well as, to a lesser extent, patient outcomes. Unlike other types of behavior change interventions, such as handouts or text messages, educational meetings are more likely to improve compliance with desired medical practice [66].

Orthopedic surgeons support the concept of opinion leaders. They believe that they can identify a small number of local colleagues with this potential, but at the national level there are clear opinion leaders. Once these are identified, Young et al. [67] propose a survey to delineate their influence in improving evidence-based surgical practices.

We used these medical practices to design the indicator EA7—Community-involved local opinion leaders (with the content shown in Table A11). With their support, the basic medical activity of local opinion leaders’ evaluation and involvement (Evaluation-A) is evaluated.

Health partnerships bring together diverse partners who propose system changes, a clear agenda, and the collection of human and financial assets that are aligned to the partnership. To ensure the satisfaction of a partnership, the participation of partners must be monitored and measured, as should the constructive collaboration and collective impact of the partnership [68]. Partnerships that aim to design and implement innovative approaches to improve population health require the continuous improvement of the partnership initiative. For this, Willis et al. [69] show the need for a shared vision, understanding the impact of a partnership and using a shared system for measuring knowledge exchange between partners.

Multisector partnership initiatives in health must start from the organizational and individual capacities needed to approach a partnership and the evaluation techniques needed to capture the effects of a partnership [70]. Guilfoyle et al. [71] identified common characteristics of successful healthcare partnerships. These are joint decisions between institutions, objectives related to education, medical training, curriculum development, securing funding for a minimum interval of 10 years, real collaboration, and the equal participation of partners.

Mudyarabikwa et al. [72] show that the success and satisfaction of public–private partnerships depend on the ability of private partners to evaluate how a partnership adds value to their own activities and the ability to align business principles with a partnership’s objectives. Public hospitals seeking to attract private funding may face challenges in addressing the material conditions of partner capacity, establishing trust, and aligning interests and motivations between partners [73].

We used these medical practices to design the indicator EB7—Satisfaction with partnerships (having the content shown in Table A13). With their support, the basic medical activity of satisfaction assessment for patients and staff (Evaluation-B) is evaluated.

#### 2.2.4. Practices for Medical Assistance Continuous Improvement

A new model of health in which several stakeholders are involved with the objective of improving the health of the community is responsible care communities. Included in these initiatives are public health organizations, medical assistance systems, and community organizations. Engaged stakeholders share resources, responsibility, and data for improving community health indicators. In this way, medical costs are reduced and economic as well as social problems that characterize the health of a population are addressed [74]. Park et al. [75] assessed the characteristics of hospitals that developed partnerships to improve population health. They conclude that large, non-profit hospitals located in congested areas have the strongest relationships.

Fields [76] opines that improvements in the health outcomes of a community are best achieved by addressing gaps in the social determinants of community members. Community-based health can also be promoted through coalition initiatives. Nagorcka-Smith et al. [77] observed statistically significant associations between a wide range of coalition characteristics and community outcomes. These include coalition structure, member characteristics, resources, community context, community partnership, communication, engagement, relationships, group dynamics, health promotion planning, and implementation.

A community initiative is a group of vulnerable communities, established during the pandemic. Gardiner and Martin [78] describe how practitioners can use a practice framework to reduce isolation and disseminate resources and information that mitigate the effects of the pandemic. Age-friendly community initiatives engage stakeholders with the goal of transforming social environments into those more conducive to health and well-being, and increasing the ability of older adults to age in the community at home [79].

We used these medical practices to design the indicator RA7—Initiatives together with the community (having the content presented in Table A15). With their support, the basic medical activity of staff self-assessment (Review-A) is evaluated.

Reckrey et al. [80] present a program of educational visits in which visiting doctors teach primary care at home for students in different medical disciplines. They show that an intern’s education and practice needs can be improved by performing independent urgent visits, assessing the health status of patients at home in collaboration with remote medical personnel, and performing subspecialty consultations. Educational visits facilitate adherence to evidence-based guidelines in primary care for knee osteoarthritis. However, the quality indicators and the probability of prescribing physical therapy recovery do not change significantly [81]. Educational visits to disseminate opioid prescribing can successfully change the prescribing behavior of recipes [82].

Mun et al. [83] describe a community-based integrated care initiative that coordinates home healthcare providers. With the support of a consortium, the integration of primary medical care–hospital–personal care–social services is achieved, and, in this way, a framework for the aging of a population at home is created. In a suburban medical center, nurses who received minimal verbal educational support through brief visits from a research nurse had significantly greater use of the center for nursing care compared to those who received services only [84].

Mahoney et al. [85] identify modifiable risk factors for minimizing postoperative costs in orthopedic surgery. They conclude that the risk of postoperative emergency department visits is associated with a lower level of education and health literacy.

We used these medical practices to design the indicator RB7—Educational visits (with the content shown in Table A17). With their support, the basic medical activity of medical services innovation (Review-B) is evaluated.

### 2.3. Indicators’ Contents and the Evaluation Grids

We developed the contents and descriptions of the 9 indicators based on proven evidence of community involvement and development activities, from Section 2.2. With this support, we developed a package of questions for the evaluation of each indicator, to cover their contents as well as possible. Next, we qualitatively described the degree of fulfillment of each indicator on the levels: not relevant, low, satisfactory, good, very good, and excellent. We also associated them with numerical values from 0 to 5.

Given that the indicators can present various levels of importance, in the evaluation system we have defined a second variable that qualitatively ranks the importance of the indicators in levels: not relevant, unimportant, reduced importance, important, very important, and high importance. Additionally, to this variable we have associated numerical values from 0 to 5. In the end, in the system designed by us, the indicators are evaluated by the pair of values of achievement degree–importance [25,26,27,28,29,30].

Considering the extensive content of the nine indicators that describe community involvement and development and the related evaluation grids, we present them in Table A1, Table A2, Table A3, Table A4, Table A5, Table A6, Table A7, Table A8, Table A9, Table A10, Table A11, Table A12, Table A13, Table A14, Table A15, Table A16, Table A17 and Table A18 of Appendix A, as follows: Table A1. The indicator PA7—Community engagement actions. Table A2. Scale for indicator PA7—Community engagement actions. Table A3. The indicator PB7—The interventions’ content adapted to the community. Table A4. Scale for indicator PB7—The interventions’ content adapted to the community. Table A5. The indicator IA71—Partnership and networking. Table A6. Scale for indicator IA71—Partnership and networking. Table A7. The indicator IA72—Involvement of volunteers and training networks. Table A8. Scale for indicator IA72—Involvement of volunteers and training networks. Table A9. The indicator IB7—Involvement and participation of professional associations. Table A10. Scale for indicator IB7—Involvement and participation of professional associations. Table A11. The indicator EA7—Community-involved local opinion leaders. Table A12. Scale for indicator EA7—Community-involved local opinion leaders. Table A13. The indicator EB7—Satisfaction with partnerships. Table A14. Scale for indicator EB7—Satisfaction with partnerships. Table A15. The indicator RA7—Initiatives together with the community. Table A16. Scale for indicator RA7—Initiatives together with the community. Table A17. The indicator RB7—Educational visits. Table A18. Scale for indicator RB7—Educational visits.

We exemplify the way in which the PA7—Community engagement actions indicator is defined in Table A1: the direct involvement of the local community in support for the provision of medical services. The questions formulated for its evaluation are as follows: Are specific regional/community requirements integrated into healthcare programs? Are new national/global technologies transferred within the regional/local program? Are regular meetings held with community representatives to exchange information and define goals? Are partnerships established with local organizations (local councils, county councils, high schools, universities, and non-profit organizations) and/or is there involvement in scientific/educational development with partners from the local community as part of the project implementation?

The evaluation scale of the indicator PA7—Community engagement actions, presented in Table A2, consists of the following scores: 1—Low: The healthcare facility has established partnerships with local organizations: the local council, the county council, high schools, universities, non-profit organizations, etc.; 2—Satisfactory: The specific regional/community requirements related to healthcare are collected; 3—Good: Specific regional/community requirements are integrated into the healthcare programs; 4—Very good: The regional/local healthcare services program transfers new national/global technologies; and 5—Excellent: Regular meetings are held with community representatives to exchange information and define goals. There is involvement in scientific/educational development with local community partners as part of project implementation.

We validated the developed theoretical model in practice by implementing it at the Orthopedics Department of the Targu Mures County Emergency Clinical Hospital (CECHM) [86]. The testing and validation in health practice of the nine indicators of the innovative Health-Sustainability reference framework was carried out through their self-evaluation at the emergency hospital in Targu Mures. The team of auditors was composed of four people working in the health and quality assurance field of the hospital: a quality assurance manager, chief assistant, and orthopedic resident physician, who were coordinated by the chief physician of the department.

**Figure 3:**
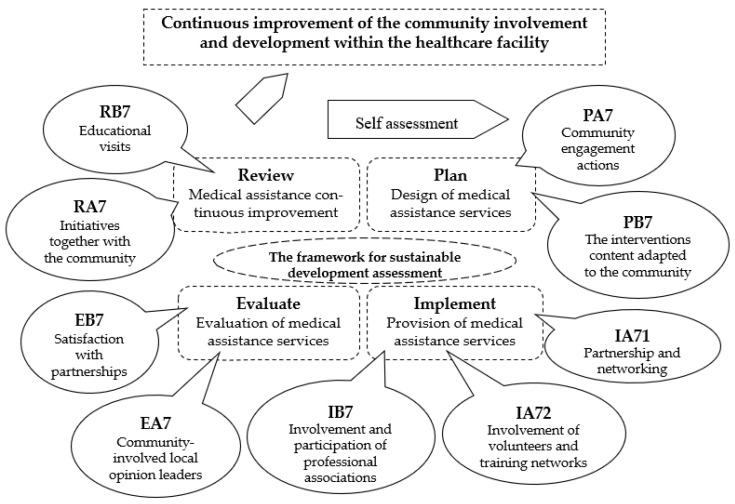
The community involvement and development continuous improvement cycle within the healthcare facility.

The implementation followed the order of the indicators designed in the stages of the continuous improvement cycle, as follows: in the planning stage it used the PA7—Community engagement actions and PB7—The interventions’ content adapted to the community indicators (Figure 3). The second stage of implementation was continued with the IA71—Partnership and networking, IA72—Involvement of volunteers and training networks, and IB7—Involvement and participation of professional associations indicators. The evaluation was conducted with the support of the EA7—Community-involved local opinion leaders and EB7—Satisfaction with partnerships indicators. At the end, the fourth review stage consisted of the use of RA7—Initiatives together with the community and RB7—Educational visits indicators.

## 3. Results

With the support of activities with evidence in the scientific literature (see Section 2.2.), the indicator matrix associated to the community involvement and development of the Health-Sustainability reference framework was projected (Table 1).

In the stage of exploring the scientific literature, we discovered some links between the basic medical activities and social responsibility of the patient. Based on them, we named the indicators and made the links between column 2 of the table, in which there are the eight basic medical activities of the quality cycle, and column 3 of the table, in which there are the social responsibility for community involvement and development indicators.

In previous research, indicator matrices and their contents are introduced in the following areas of social responsibility: organizational governance [25], human rights [26], labor practices [27], the environment [28], fair healthcare practices [29], and patient matters [30]. In this research, the community involvement and development area of social responsibility and the nine indicators that make it up are detailed.

By testing the nine indicators of community involvement and development at the emergency hospital in Targu Mures, we obtained the results, which are briefly presented in the continuation of this section.

PA7—Community engagement actions: The hospital has signed collaboration contracts with the Mures County Council, the General Directorate of Social Assistance and Child Protection Mureș, the “Together for Children with Cancer” Association, the Oncological Institute “Prof. Dr. Al. Trestioreanu” Bucharest, the Mures County Directorate for the Registration of Persons, the National Anti-Drug Agency, the National Register of Voluntary Donors of Hematopoietic Stem Cells, and the Inspectorate for Emergency Situations “Horea” of Mures County, some of them within the framework of educational projects. Patient associations are involved in the hospital’s Ethical Council.

PB7—The interventions’ content adapted to the community: The hospital has adopted the objectives of community medical assistance, to identify in collaboration with the social assistance service the medico-social problems of the community and vulnerable groups, facilitating their access to healthcare services, implementing public healthcare programs and actions continuously adapted to the needs of the community, participating in collective medical actions in the community (e.g., screening programs, vaccinations), making home visits, providing emergency medical assistance services, medical and social counseling.

IA71—Partnership and networking: The hospital collaborates with other healthcare facilities to provide medical assistance, being functionally integrated with other health units in the public health network. For carrying out the activity of providing emergency medical assistance on site, taking over the patient and transporting to the Emergency Reception Unit (ERU), the hospital benefits from the support of the “Horea” Emergency Situations Inspectorate of Mureș County. The collaboration between the two institutions is based on collaboration protocols and agreements. On the administrative board consisting of five members, there is a representative of the Mureș County Council.

IA72—Involvement of volunteers and training networks: Volunteers are mainly involved in the support activities of the Emergency Reception Unit. ERU-SMURD Targu-Mures, in collaboration with the Emergency Medicine Student Organization, organizes courses for beginner volunteers. Online training modules are organized, through which the Foundation for SMURD Targu Mures facilitates the training process of volunteers through educational content in the form of text, documents, multimedia elements, links, tests, and questions. The internships organized for volunteers and how the hospital develops and coordinates volunteering in orthopedics–traumatology were not highlighted.

IB7—Involvement and participation of professional associations: The smooth operation and carrying out of the transfer activity within the hospital is ensured by the activity of the transport office. It has eight functional vehicles of different types that allow transfers to hospital structures of patients, staff, and products. Emergency interventions by UPU-SMURD are carried out with a number of 25 vehicles, as well as with vehicles of different types from the First Aid Teams from Mures county—Deda, Ibanesti, Iernut, Sovata, Miercurea Nirajului, Acatari, Raciu, and Ludus, as well as with the ambulances taken over on loan based on the collaboration contracts with ISU “Horea” Mures.

EA7—Community-involved local opinion leaders: Local opinion leaders are part of the community working groups of the Municipal Council and the County Council. There are local initiatives to improve the medical infrastructure, some of which are supported by sponsorships (e.g., Romgaz). They participate in various national or international courses and congresses, some of them supported by companies producing prostheses.

EB7—Satisfaction with partnerships: A comprehensive assessment of partnership satisfaction is possible as a result of combining data sources. The main source of data collection is the discussions with the members of the partnership coordination team, the interviews with stakeholders at the community level, and the managers of the partner organizations, as well as the analysis of the qualitative and quantitative secondary data, collected with the support of the patient satisfaction questionnaires.

RA7—Initiatives together with the community: These initiatives consist of integrated community-based services (medical [87], social [88,89], and educational [90,91,92]), which are delivered by a community-based team. These usually involve a thorough assessment of family members, a shared location, or are based on case management.

RB7—Educational visits: With the support of the University of Medicine “G.E.Palade” from Targu Mures, presentations of medical personalities are organized. These aim to improve current professional practices in the hospital. Postgraduate courses are held for residents, but also for different categories of doctors. Personalities participate in the presentation of the doctorate theses of the teaching staff of the hospital.

The values computed for the indicators related to community involvement and development responsibility are included in the self-assessment tool (Table 2).

**Figure 4:**
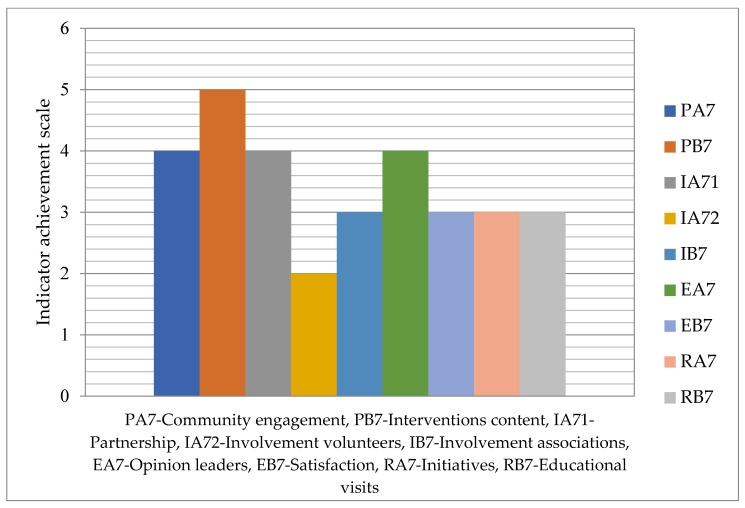
Achievement degree for community involvement and development responsibility.

In Figure 4, on a scale in the range 1–5, the degree achievement of indicators related to community involvement and development is indicated. In this domain, the indicator IA72—Involvement of volunteers and training networks has reached the minimum value, 2, while the highest value, 5, is noted for the indicator PB7—The interventions’ content adapted to the community.

**Figure 5:**
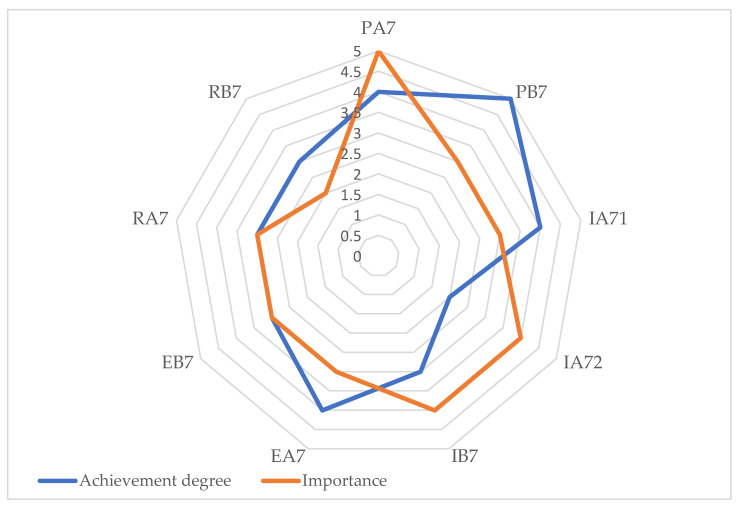
The community involvement and development evaluation graph: PA7—Community engagement actions; PB7—The interventions’ content adapted to the community; IA71—Partnership and networking; IA72—Involvement of volunteers and training networks; IB7—Involvement and participation of professional associations; EA7—Community-involved local opinion leaders; EB7—Satisfaction with partnerships; RA7—Initiatives together with the community; and RB7—Educational visits.

The evaluation graph in Figure 5 depicts the correlation between the achievement degree and the importance of the indicators related to community involvement and development.

By summing up the values of individual community involvement and development indicators in Table 2, the global sustainability indicator (GS_CID_) is deduced as follows:

(1) $${\mathrm{GS}}_{\mathrm{CID}}=\overset{9}{\underset{i=1}{\sum }}S_{i}=\overset{9}{\underset{i=1}{\sum }}I_{i}\cdot A_{i}=103$$

The maximum value of global sustainability for the community involvement and development is the sum of the maximum values of the indicators that make it up (GSmax_CID_):

(2) $${\mathrm{GSmax}}_{\mathrm{CID}}=5\times \overset{9}{\underset{i=1}{\sum }}I_{i}=5\times 30=150$$

The ratio between the current value and the maximum value of the indicator reflects the overall community involvement and development sustainability level (LGS_CID_):

(3) $${\mathrm{LGS}}_{\mathrm{CID}}=\frac{{\mathrm{GS}}_{\mathrm{CID}}}{{\mathrm{GSmax}}_{\mathrm{CID}}}\times 100=\frac{103}{150}\times 100=68.66\%$$

**Figure 6:**
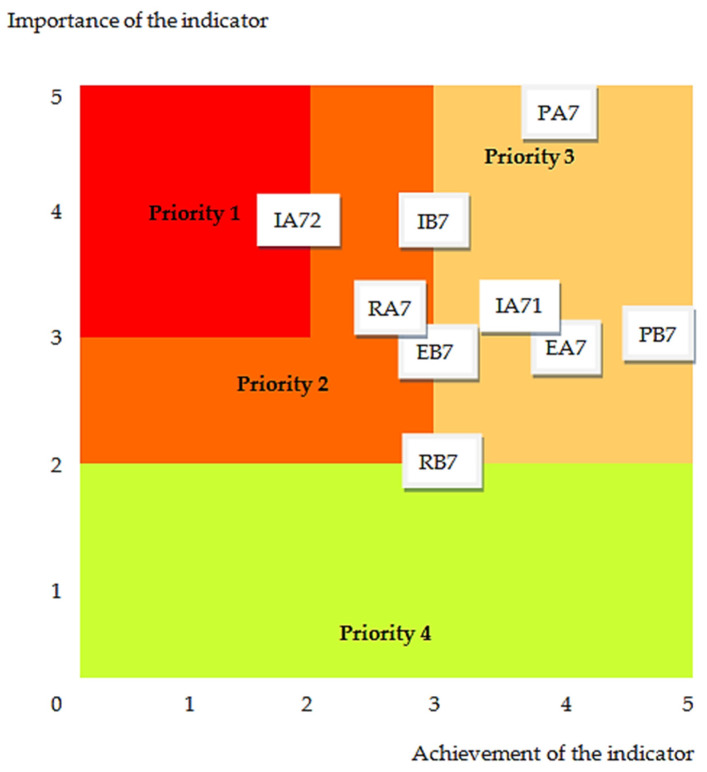
The assessment diagram for community involvement and development: PA7—Community engagement actions; PB7—The interventions’ content adapted to the community; IA71—Partnership and networking; IA72—Involvement of volunteers and training networks; IB7—Involvement and participation of professional associations; EA7—Community-involved local opinion leaders; EB7—Satisfaction with partnerships; RA7—Initiatives together with the community; and RB7—Educational visits.

The sustainability level reflects the extent to which the social requirements of community involvement and development are met by the hospital where we performed the evaluation. To increase this level, with the support of the Eisenhower-type matrix in Figure 6, we ranked the order of implementation of the indicator’s improvement measures. In its field, the importance and urgency of subsequent actions are represented between the extremes of high priority (1) and low priority (4).

In the case of the current evaluation at the emergency hospital in Targu Mures, for the short-term improvement of the social responsibility related to community involvement and development, it is necessary to give the highest priority to the indicator IA72—Involvement of volunteers and training networks.

## 4. Discussion

Following the evaluation of the indicators, we made a first finding of our study, namely the adequacy of the designed indicators with the proposed purpose of the research. We appreciated that they are useful in evaluating the social responsibility requirement related to community involvement and the development of sustainable medical services. We also found that the new tool created through its indicators responds to the secondary purpose of the research to ensure compatibility with the other reference frameworks implemented in the hospital. It is compatible with the requirements of the national legislation for the accreditation of outpatient healthcare services [93] as well as the requirements of the national legislation for the accreditation of healthcare facilities with beds [94], but also with the requirements of the European DUQuE hospital quality assessment framework [95].

As regards indicators’ contents, we also found that they can be better adapted to the particularities of the evaluated healthcare facility. For this reason, hospitals that adopt the proposed methodology should customize the content of indicators and evaluation grids [25]. This would also facilitate the tracking of progress in the degree of fulfillment of the indicators [27]. The development of a glossary with the specific terminology would facilitate a good understanding of the reference framework among the members of the evaluation team but also between different evaluation teams and the evaluated. In this way, an even better objectivity of evaluations would be facilitated through the mutual understanding of terminology as stated also by Heinemeyer et al. [96].

Other findings relate to audit planning and the human resources involved. We found, in accordance with Wang et al. [97], that there is a need for good communication with representatives of the audited departments and the scheduling of the audit in a reasonable time frame in which they can prepare the audit evidence in advance, but also be able to present and support it at the audit. For this reason, the chief auditor must be a person with authority, who commands respect from the auditees, and who rigorously plans as well as organizes the assessment mission [29].

Regarding the sustainability aspects, similar to Montague et al. [46] we found that the implementation of the new reference framework contributed to the increase in the social responsibility of the health personnel in terms of community involvement and development in the current activities of the hospital [26]. With their support, the administrative and sanitary practices that have a significant contribution to the sustainable development of the hospital will be periodically evaluated [30].

Another finding of our study indicates that the indicator IA72—Involvement of volunteers and training networks must be treated with priority in order to bring about a significant improvement in the hospital’s sustainability in the short term. Thus, together with educational and professional networks, the hospital must carry out regular activities and internships in orthopedics–traumatology for volunteers. The hospital must support and develop volunteerism permanently in order to increase its contribution to the development of the quality of healthcare. Quality volunteering in the area of care and competence should be supported, while optimizing mechanisms for the effective coordination of volunteers.

Similar to the study by Gholipour et al. [14], we found the existence of some barriers in the institutionalization of community participation within the hospital. A major problem in the way of participation is the community’s trust in the health system due to previous less successful programs. Also, the reluctant attitude of healthcare managers towards community participation reduces the degree of involvement of citizens in social processes. But, unlike them, we found that the adequate planning of healthcare processes facilitates community participation in pilot healthcare programs.

Consistent with the results reported by Sacks et al. [18], in our study we found that increasing patients’ trust in the health system and promoting its efficiency can lead to community participation in the provision of healthcare.

Unlike Ayton et al. [16], we can say that cultivating the trust of health personnel at the organizational level can act as a “social binder” that can bring together several organizations in collaboration with the hospital. Ignoring this principle demotivates hospital employees to have a participatory attitude and cooperate with other structures. This results in substandard performance.

We also found that managers of healthcare facilities should continuously monitor the state of public confidence in the health system. In launching healthcare programs, community expectations should first be examined and then community involvement in planning. The weak position of community representation in the high-level decision-making process, but also in the programs coordinated by the Ministry of Health at the national level, generates a weak organization by program managers in terms of community participation. It would be necessary to establish a specific structure to attract community participation in the healthcare system, as recommended in this study and Eftekhari et al. [98].

Consistent with the results reported by Rangan et al. [55], we found that there are collaborations of medical staff with networks that support clinical care. The use of networks has been effective in conducting clinical trials on increasing the efficiency of preoperative planning in tibial plateau fractures with the help of custom surgical instruments built by 3D technologies [99], but this required some investment to build research capacity in medical robotics [100].

According to the findings of the current study, we found the existence of financial relationships between the representatives of the companies producing prostheses and the orthopedics–traumatology medical association, which aim to improve medical care. One concern is continuing medical education by participating in various national or international courses and congresses that are funded by these companies. Unlike Schofferman [57], we could not assess whether these relationships, by their nature, present conflicts of interest and detect a bias in medical decisions.

In the hospital, we found that medical staff support the role of opinion leaders. However, we identified a small number of people with this potential, using certain medical techniques. Contrary to the conclusions formulated by Young et al. [57], within the hospital there is no availability for the organization of surveys through which it is possible to delineate their influence in improving evidence-based surgical practices.

There are some limitations in the study we conducted. It is possible that, in the stage of database exploration, some successful medical activities were missed that could have contributed to a better design of the indicators. Also, we mainly searched for information about the orthopedics–traumatology specialty, and, by searching for other keywords, relevant information can also be found for other medical specialties that can enrich the contents of the indicators. Additionally, the validation of the indicators in practice was carried out in an emergency hospital in the orthopedics–traumatology specialty. This constitutes another limitation of the study, and, through validations in other medical specialties or hospitals with other forms of organization and ownership than a state one, greater adaptation and generalization of the indicators would be obtained.

The directions for future research are results of the identified limitations. We propose to continue the study through recent searches in the databases followed by adapting the indicators’ contents to respond to as many healthcare and social requirements as possible. Additionally, the digitization process of the indicators would facilitate the evaluation and ensure its traceability, as support for the continuous improvement processes of healthcare facilities.

## 5. Conclusions

In this research, we developed nine indicators with the support of which healthcare facilities can evaluate community investment and development as part of the processes of social responsibility and sustainable development. The content of the indicators is developed based on collaborative activities between hospitals and the community, which are described in the scientific literature as successful. We made a description of the indicators and developed a set of questions for their evaluation. We developed an innovative format for evaluating the indicators, which is made up of the information couple degree of achievement–importance. Each of these are evaluated qualitatively but also numerically by values from 0 to 5. The easy framing of the indicators is carried out with the support of the evaluation grids, which describe the fulfillment requirements at each step. This evaluation mode allows the prioritization of improvement measures and the degree of the sustainable development of the evaluated healthcare facility, supported by an Eisenhower-type matrix.

The validation of the indicators in practice at an orthopedic specialty emergency hospital revealed their suitability for the intended purpose and compatibility with the national requirements for the accreditation of hospitals with beds and of ambulatory units, as well as with the European framework for the quality evaluation of hospitals. Through its indicators, the new reference framework guides the health staff, patients, the hospital, staff from collaborating organizations, and community organizations in collaboration with the hospital towards sustainability. This constitutes another of its attributes in relation to the requirements applied by hospitals today.

## Figures and Tables

**Table 1:** Community involvement and development indicator matrix of the H-S framework.

Quality Cycle		Community Involvement and Development—Social Responsibility
(Plan) Design of medical assistance services	Plan-A Hospital institutional accreditation	PA7—Community involvement activities
	Plan-B Designing patient-oriented medical services	PB7—Content of the interventions adapted to the community
(Implement) Provision of medical assistance services	Implement-A Provision of medical services	IA71—Networking and partnership IA72—Involvement of volunteers and training networks
	Implement-B Patient transfer provision	IB7—Involvement and participation of professional associations
(Evaluate) Evaluation of medical assistance services	Evaluation-A Local opinion leaders’ evaluation and involvement	EA7—Local opinion leaders involved in the community
	Evaluation-B Satisfaction assessment for patients and staff	EB7—Satisfaction regarding partnerships
(Review) Medical assistance continuous improvement	Review-A Staff self-assessment	RA7—Communitarian initiatives
	Review-B Medical services innovation	RB7—Educational visits

**Table 2:** Self-assessment tool for community involvement and development responsibility.

No.	Indicator Descriptive	Importance (Ii)	Achievement (Ai)	Sustainability Indicator (Si = Ii·Ai)
1	PA7—Community engagement actions	5	4	20
2	PB7—The intervention’s content adapted to the community	3	5	15
3	IA71—Partnership and networking	3	4	12
4	IA72—Involvement of volunteers and training networks	4	2	8
5	IB7—Involvement and participation of professional associations	4	3	12
6	EA7—Community-involved local opinion leaders	3	4	12
7	EB7—Satisfaction with partnerships	3	3	9
8	RA7—Initiatives together with the community	3	3	9
9	RB7—Educational visits	2	3	6

Ii—importance, Ai—achievement, and Si—sustainability indicator.

## Data Availability

The data used in this study can be requested from the corresponding author.

## Appendix A

**Table A1:** The indicator PA7—Community engagement actions.

Indicator	PA7—Community Engagement Actions
Description	Direct involvement of the local community in support of the provision of medical services.
Evaluation question	Are specific regional/community requirements integrated into healthcare programs? Are new national/global technologies transferred within the regional/local program? Are regular meetings held with community representatives to exchange information and define goals? Are partnerships established with local organizations (local councils, county councils, high schools, universities, and non-profit organizations) and/or is there involvement in scientific/educational development with partners from the local community as part of project implementation?

**Table A2:** Scale for indicator PA7—Community engagement actions.

Score [A]	Achievement	Content
0	Not relevant	-
1	Low	The healthcare facility has established partnerships with local organizations: the local council, the county council, high schools, universities, non-profit organizations, etc.
2	Satisfactory	The specific regional/community requirements related to healthcare are collected.
3	Good	Specific regional/community requirements are integrated into the healthcare programs.
4	Very good	Regional/local healthcare services program transfers new national/global technologies.
5	Excellent	Regular meetings are held with community representatives to exchange information and define goals. There is involvement in scientific/educational development with local community partners as part of project implementation.

**Table A3:** The indicator PB7—The interventions content adapted to the community.

Indicator	PB7—The Interventions’ Content Adapted to the Community
Description	The content of patient-centered care takes into account social evolution: – The degree of population aging; – The prevalence of certain conditions.
Evaluation questions	Is the social evolution of patients an input to the design of patient-centered care? Does the content of patient-centered care take into account the aging of the population? Does the content of patient-centered care take into account the prevalence of certain conditions?

**Table A4:** Scale for indicator PB7—The interventions’ content adapted to the community.

Score [A]	Achievement	Content
0	Not relevant	-
1	Low	Information is collected regarding social aspects, the prevalence of certain conditions, the degree of aging of the population in the community.
2	Satisfactory	The social evolution of patients is an input element in the design of patient-centered care.
3	Good	The content of patient-centered care takes into account the aging of the population.
4	Very good	The content of patient-centered care takes into account the prevalence of certain conditions.
5	Excellent	The content of medical interventions is permanently adapted to the community according to the identified requirements.

**Table A5:** The indicator IA71—Partnership and networking.

Indicator	IA71—Partnership and Networking
Description	Collaboration between healthcare facilities to ensure medical assistance. Involvement of municipalities, governmental/local representative institutions in the decision-making and support processes. Using municipal facilities and assets to support healthcare.
Evaluation questions	Does the organization collaborate with other healthcare facilities to provide medical assistance? Are the municipality, government/local representative institutions involved in the decision-making and support processes? Does the municipality’s heritage provide support for healthcare?

**Table A6:** Scale for indicator IA71—Partnership and networking.

Score [A]	Achievement	Content
0	Not relevant	-
1	Low	There is concern for networking with other healthcare facilities and partnerships with other institutions.
2	Satisfactory	The organization collaborates with other healthcare facilities to provide medical assistance.
3	Good	The organization is functionally integrated with other healthcare facilities, with roles and functions specific to each level of complexity and competence of medical assistance.
4	Very good	The municipality, government/local representative institutions are involved in the decision-making and support processes of the healthcare facility.
5	Excellent	The healthcare facility offers medical assistance in which support is also provided by the municipality’s heritage.

**Table A7:** The indicator IA72—Involvement of volunteers and training networks.

Indicator	IA72—Involvement of Volunteers and Training Networks
Description	Involvement of volunteers in support activities. Regular activities with educational and professional networks (e.g., practice).
Evaluation questions	Are volunteers involved in support activities? Are there regular educational and professional networking activities? For example, practice is evaluated.

**Table A8:** Scale for indicator IA72—Involvement of volunteers and training networks.

Score [A]	Achievement	Content
0	Not relevant	-
1	Low	The healthcare facility has initiated a volunteering program and assumes the responsibility of recognizing the value of volunteering.
2	Satisfactory	Volunteers are involved in the support activities of the healthcare facility.
3	Good	The healthcare facility carries out regular activities with educational and professional networks in order to train volunteers. Internships are held.
4	Very good	The healthcare facility supports and develops volunteerism in order to increase its contribution to the development of the quality of healthcare.
5	Excellent	The healthcare facility supports quality volunteering, especially in the area of care and competence, assuming the duty to offer a quality volunteering experience, through the effective coordination of volunteers.

**Table A9:** The indicator IB7—Involvement and participation of professional associations.

Indicator	IB7—Involvement and Participation of Professional Associations
Description	Professional associations can support discharge planning by developing guidelines for the transfer of critically ill patients.
Evaluation questions	Are professional associations involved in discharge planning? Do professional associations contribute to the development of guidelines for the transfer of critically ill patients?

**Table A10:** Scale for indicator IB7—Involvement and participation of professional associations.

Score [A]	Achievement	Content
0	Not relevant	-
1	Low	The healthcare facility has contacts with professional associations of doctors, nurses, patient associations, etc.
2	Satisfactory	Patient discharge is planned and coordinated with the support of professional associations.
3	Good	Upon discharge, professional associations contribute to informing patients about possible errors that may occur in the administration of high-risk drugs, by providing written materials that can also be used after discharge.
4	Very good	Professional associations contribute to the collection of discharge information and forward it to the healthcare facility.
5	Excellent	Professional associations contribute to the development of guidelines for the transfer of critically ill patients.

**Table A11:** The indicator EA7—Community-involved local opinion leaders.

Indicator	EA7—Community-Involved Local Opinion Leaders
Description	Local opinion leaders are involved in the following: – Community awareness activities; – Community working groups.
Evaluation questions	Are local opinion leaders involved in community outreach activities? Do local opinion leaders participate in community working groups?

**Table A12:** Scale for indicator EA7—Community-involved local opinion leaders.

Score [A]	Achievement	Content
0	Not relevant	-
1	Low	Local opinion leaders have contacts with professional associations and community organizations.
2	Satisfactory	Local opinion leaders are involved in community outreach activities.
3	Good	Local opinion leaders participate in community working groups.
4	Very good	There are suggestions for improvements made by working groups of local opinion leaders and community organizations.
5	Excellent	The suggestions for improvement formulated by the working groups constituted by local opinion leaders and community organizations are transposed into the practice of the healthcare facility.

**Table A13:** The indicator EB7—Satisfaction with partnerships.

Indicator	EB7—Satisfaction with Partnerships
Description	The measure of patient satisfaction regarding the involvement and participation of partners and stakeholders, governmental, and non-governmental organizations, such as the Directorate of Public Health, the National Health Insurance House, high schools, and universities, regarding the following: (1) Care at home; (2) Assistance to people with vulnerabilities, etc.
Evaluation questions	Does the organization have partners, stakeholders, governmental and non-governmental organizations such as: Public Health Directorate, National Health Insurance House, high schools, and universities? Is patient satisfaction measured regarding partners’ involvement and participation in home care? Is patient satisfaction measured in terms of partners’ involvement and participation in assisting people with vulnerabilities?

**Table A14:** Scale for indicator EB7—Satisfaction with partnerships.

Score [A]	Achievement	Content
0	Not relevant	-
1	Low	The healthcare facility has partners, stakeholders, governmental, and non-governmental organizations, such as the Public Health Directorate, the National Health Insurance House, high schools, and universities.
2	Satisfactory	There are updated questionnaires to assess patient satisfaction with partner involvement and participation in home care.
3	Good	Patient satisfaction with partner involvement and participation in home care is measured.
4	Very good	Patient satisfaction with partners’ involvement and participation in assisting people with vulnerabilities is measured.
5	Excellent	Based on the evaluation results, improvement measures are established to increase patient satisfaction.

**Table A15:** The indicator RA7—Initiatives together with the community.

Indicator	RA7—Initiatives Together with the Community
Description	Measuring the number of initiatives taken with community engagement.
Evaluation questions	Are community-engaging initiatives implemented? If so, how many initiatives are implemented annually? Are existing community initiatives evaluated and ways of improvement identified?

**Table A16:** Scale for indicator RA7—Initiatives together with the community.

Score [A]	Achievement	Content
0	Not relevant	-
1	Low	The healthcare facility is involved in community initiatives.
2	Satisfactory	Initiatives involving the community are implemented.
3	Good	There are a considerable number of initiatives with the community that are being implemented.
4	Very good	Periodically, existing community initiatives are evaluated and ways to improve them are identified.
5	Excellent	Identified improvement measures are implemented.

**Table A17:** The indicator RB7—Educational visits.

Indicator	RB7—Educational Visits
Description	Educational visits are quality improvement interventions aimed at improving professional healthcare practices and health outcomes. Trained people visit the healthcare facility and provide information to improve current practices. The information provided may include feedback on one’s own performance or deal with ways to overcome obstacles to planned change. The qualifications of the visitor carrying out the educational visits are important for efficiency.
Evaluation questions	Are educational visits organized to improve current practices? What is the qualification level of those who carry out educational visits? Does the information submitted show the performance of those supporting the visits? Are ways to overcome obstacles to planned change presented?

**Table A18:** Scale for indicator RB7—Educational visits.

Score [A]	Achievement	Content
0	Not relevant	-
1	Low	The healthcare facility contacts medical professionals in order to organize educational visits.
2	Satisfactory	Educational visits are organized to improve current professional healthcare practices and health outcomes.
3	Good	Trained people visit the healthcare facility and provide information to improve current practices.
4	Very good	The information provided includes feedback on the performance of those supporting the visits.
5	Excellent	The information provided deals with ways to overcome obstacles that arise in the way of planned changes.
